# Persistent reduction in sialylation of cerebral glycoproteins following postnatal inflammatory exposure

**DOI:** 10.1186/s12974-018-1367-2

**Published:** 2018-12-05

**Authors:** Ekaterina P. Demina, Wyston C. Pierre, Annie L. A. Nguyen, Irene Londono, Bela Reiz, Chunxia Zou, Radhika Chakraberty, Christopher W. Cairo, Alexey V. Pshezhetsky, Gregory A. Lodygensky

**Affiliations:** 1 Department of Paediatrics, Sainte-Justine Hospital Research Center, Université de Montréal, Montreal, H3T 1C5 QC Canada; 2 Alberta Glycomics Centre and Department of Chemistry, University of Alberta, Edmonton, T6G 2G2 AB Canada; 3 Department of Anatomy and Cell Biology, McGill University, Montreal, H3A0C7 QC Canada; 4 Department of Pharmacology and Physiology, Université de Montréal, Montreal, H3T 1J4 QC Canada; 5 Montreal Heart Institute, Montreal, H1T 1C8 QC Canada; 6 Centre de recherche, CHU Sainte-Justine, 3175 Côte-Sainte-Catherine, Montreal, QC H3T 1C5 Canada

**Keywords:** Lysosomal dysfunction, Neonatal neuroinflammation, Neuronal neuraminidase 1, Neonatal rat model, Sialic acid

## Abstract

**Background:**

The extension of sepsis encompassing the preterm newborn’s brain is often overlooked due to technical challenges in this highly vulnerable population, yet it leads to substantial long-term neurodevelopmental disabilities. In this study, we demonstrate how neonatal neuroinflammation following postnatal *E. coli* lipopolysaccharide (LPS) exposure in rat pups results in persistent reduction in sialylation of cerebral glycoproteins.

**Methods:**

Male Sprague-Dawley rat pups at postnatal day 3 (P3) were injected in the corpus callosum with saline or LPS. Twenty-four hours (P4) or 21 days (P24) following injection, brains were extracted and analyzed for neuraminidase activity and expression as well as for sialylation of cerebral glycoproteins and glycolipids.

**Results:**

At both P4 and P24, we detected a significant increase of the acidic neuraminidase activity in LPS-exposed rats. It correlated with significantly increased neuraminidase 1 (*Neu1*) mRNA in LPS-treated brains at P4 and with neuraminidases 1 and 4 at P24 suggesting that these enzymes were responsible for the rise of neuraminidase activity. At both P4 and P24, sialylation of N-glycans on brain glycoproteins decreased according to both mass-spectrometry analysis and lectin blotting, but the ganglioside composition remained intact. Finally, at P24, analysis of brain tissues by immunohistochemistry showed that neurons in the upper layers (II–III) of somatosensory cortex had a reduced surface content of polysialic acid.

**Conclusions:**

Together, our data demonstrate that neonatal LPS exposure results in specific and sustained induction of Neu1 and Neu4, causing long-lasting negative changes in sialylation of glycoproteins on brain cells. Considering the important roles played by sialoglycoproteins in CNS function, we speculate that observed re-programming of the brain sialome constitutes an important part of pathophysiological consequences in perinatal infectious exposure.

## Background

Sialoglycoconjugates (SGC) are found in abundance on the surface as well as on the inner sides of lysosomal and endosomal membranes of mammalian cells, forming a dense and diverse array of complex sialylated glycans, referred to as the sialome [1]. The SGC are not evenly distributed on the membrane, but rather form dynamic domains enriched in gangliosides and sialylated membrane proteins [2–7]. In mammals, the content of SGC strongly depends on the cell and tissue type and significantly changes during development [7]. Due to their diverse physical and chemical properties, sialic acids are involved in a surprising variety of biological processes mainly by modulating recognition events during cell differentiation, interaction, migration, and adhesion (reviewed in [8–10]. Glycosynapses mediate cell signaling and participate in processes such as cell adhesion, motility, and growth (reviewed in [6]).

In the central nervous system, two types of SGC, gangliosides and proteins containing polysialic acid (PSA), play determining roles (reviewed in [11]). The gangliosides are essential for myelination, neuritogenesis, synaptic plasticity, and transmission of nervous impulses. They associate laterally with membrane proteins, including receptors and ion channels, to modulate their activities. Ganglioside glycans extend into the extracellular space and interact with glycan-binding proteins to mediate cell-protein and cell-cell interactions. As for PSA, they are attached to a number of brain glycoproteins, including the neural cell adhesion molecule (NCAM), and have been implicated in the control of cell-cell and cell-matrix interactions, essential for appropriate brain development, adult brain plasticity, and nerve regeneration. Dysregulation of PSA has been associated with multiple psychiatric disorders, including schizophrenia, bipolar disorder, and autism spectrum disorders [12, 13].

Infection during the perinatal period is associated with substantial long-term neurodevelopmental disabilities [14, 15], especially in the case of meningeal involvement [16, 17] with evidence on MRI of direct white matter injury [18]. Due to the lack of physiological stability in vulnerable preterm infants, lumbar puncture is performed in only 20% of neonatal sepsis [19, 20], so that the true incidence of such cases is not available. In line with this frequent clinical situation, a robust model of inflammatory brain injury in the immature rats has been developed using an injection of lipopolysaccharide (LPS) into the corpus callosum [21–25]. Interestingly, our preliminary data showed high resemblance between the pathological changes observed in LPS-treated neonatal rat brains and neurological lysosomal storage disorders (LSD), progressive childhood diseases caused by inborn errors of catabolism. Multiple studies in animal models of LSD demonstrated that lysosomal storage of undegraded macromolecules (especially glycans and gangliosides caused by deficiencies of glycosidases) induced neuroinflammation by activating Toll-like receptors (TLR) of microglia cells (reviewed in [26, 27]). This results in the release of cytokines such as TNF-α and MIP-1-α known to cause mitochondrial damage in neurons through formation of reactive oxygen species and oxidative stress [28–31] and eventually leads to neuronal death. We hypothesized that exposure of a neonatal brain to proinflammatory compounds could cause a similar focal response.

The aim of the current study was to characterize changes in lysosomal catabolism of glycans in the neonatal brain during and after the acute inflammation period. Unexpectedly, we observed that neonatal inflammation resulted in a significant sustained increase of neuronal neuraminidase 1 (NEU1) expression and activity with no apparent differences for other lysosomal enzymes and proteins. NEU1 induction was associated with the desialylation of glycoproteins on cortical brain cells (including polysialylated neuronal glycoproteins) potentially leading to long-term alterations of neuronal plasticity and CNS homeostasis.

## Materials and methods

### Animals

Sprague-Dawley male rats (Charles River, QC, Canada) and *Hgsnat-Geo* male mice (the mouse model of human lysosomal neurological disease Mucopolysaccharidosis IIIC [32]) were maintained at the Canadian Council on Animal Care (CCAC)-accredited animal facilities of the Montreal Heart Institute and of the CHU Sainte-Justine Research Centre, respectively, according to the CCAC guidelines. Animals were housed in an enriched environment, with nesting materials, chew toy and a black cylinder open at both ends, with continuous access to food and water, under constant temperature and humidity, on a 12-h light/dark cycle.

Each rat in a litter was randomly assigned to either saline or LPS groups. Three days postnatal (P3) pups (*n* = 22) were anesthetized with isoflurane and injected under ultrasound guidance with 0.5 μL of LPS (1 mg/mL) from *Escherichia coli* 055:B5 (Sigma, St Louis, MO) suspension prepared in sterile saline. The pups in the Sham group (*n* = 15) were injected with the same volume of sterile saline. The injection was placed in the left corpus callosum at the level equivalent to P-7, c9 (Ramachandra RST, 2011, Atlas of the Neonatal Rat Brain, CRC Press, Boca Raton, FL). Injections were made with a micropipette mounted on a microprocessor-controlled injector (Nanoliter 2000; World Precision Instruments, Aston, Stevenage, UK). To increase the efficiency of the intervention, the injections were visualized by ultrasonography (Vevo LAZR imaging system, Fujifilm VisualSonics, Canada) [25]. Of 22 male pups injected with LPS, 2 died; all 15 pups injected with saline alone survived. Rats were kept with dams until brains were removed and processed for studies.

For RNA extraction, enzyme activity assays, and lectin blots, rat pups were sacrificed by overdose of isoflurane and transcardially perfused with saline. Twenty-four hours (P4) or 21 days after the injection (P24), the brains were dissected in halves and the left and right hemispheres were frozen in liquid nitrogen.

To collect brains for cryosectioning, rat pups were deeply anesthetized with isoflurane and transcardially perfused with saline, followed by phosphate-buffered 4% paraformaldehyde. The brains were immersed in paraformaldehyde for 24 h, transferred to 30% sucrose solution and kept at 4 °C before embedding with Optimal Cutting Temperature (OCT) compound at − 20 °C.

For the mouse experiment, wild-type C57Bl6 mice and previously described *Hgsnat-Geo* mice (the mouse model of lysosomal storage disease mucopolysaccharidosis IIIC [32]) were kept at the animal facility of CHU Sainte-Justine Research Center until the sacrifice at the age of 8 months; their right brain hemispheres were dissected and frozen for enzyme activity assays.

### Enzyme activity assays in brain homogenates

The right hemispheres of rat brains were coronally dissected into blocks of tissue, containing areas contralateral to the injection site 1 to − 1.80 mm from the bregma at P4 and 1 to − 4.16 mm from the bregma at P24. The tissue blocks of rat brain or whole hemispheres of mouse brains were homogenized in water using a motorized pestle.

Enzymatic assays of s*ialidase*, β-galactosidase, and β-hexosaminidase activities in brain homogenates were performed according to the previously described protocols [33]. Protein concentration was measured using a Bradford kit (Bio-Rad laboratories, Mississauga, ON, Canada).

### Quantitative RT-PCR

In rats, quantification of *Neu1*, *Neu3*, and *Neu4* mRNA expression in the left brain hemispheres was performed using the LightCycler 96 system (Roche Life Science). Brains were extracted, and the ipsilateral hemisphere was snap-frozen on dry ice. The brain tissue adjacent to the point of injection, − 1 to − 1.80 mm from the bregma at P4 and 1 to − 4.16 mm from the bregma at P24, was reduced to powder by grinding in liquid nitrogen. Total RNA was extracted with the RNeasy mini-kit according to the manufacturer’s instructions (Qiagen, Hilden, Germany). RNA quality and concentration were assessed by spectrophotometry using the Nanodrop apparatus (Thermo Scientific, Wilmington, DE, USA). Total RNA (1 μg) was subjected to reverse transcription using the iScript™ cDNA synthesis kit (Bio-Rad, Hercules, CA, USA). RT-qPCR was performed in triplicate for each sample using SYBR Green Supermix (Bio-Rad, Hercules, CA, USA) for 40 cycles with a three-step program (20 s of denaturation at 95 °C, 30 s of annealing at 58 °C, and 30 s of extension at 72 °C) using the primers indicated in Table 1. Amplification specificity was assessed with a melting curve analysis. The fold-change expression was determined by the comparative cycle threshold (CT) method (2^−ΔΔCT^) and normalized to the housekeeping gene *Gapdh*.

**Table 1:** List of primers used in quantitative RT-PCR analysis, antibodies and lectins used in the experiments and their working dilutions

Gene	Forward (5′ to 3′)	Reverse (5′ to 3′)	Reference
*Gapdh*	AAGGTCGGTGTGAACGGATT	TGAACTTGCCGTGGGTAGAG	[63]
*Neu1*	GTAGCACGTGGTCCTCTACG	ATCGTGATCGTGTTTGGGCT	Primer Blast
*Neu3*	CTCAGTCAGAGATGAGGATGCT	GTGAGACATAGTAGGCATAGGC	[64]
*Neu4*	CCGGACTGTGGTTGGTAGAC	ACCAGGGAGCGTAAAGCAAT	Primer Blast
Antibody/lectin		Working dilution	Catalog number, company
Anti GFAP (GA5), mouse mAb		1:300	3670, Cell Signaling Technology
Anti-PSA, mouse mAb		1:600	[65]
Anti-NeuN, mouse mAb		1:200	MAB377, EMD Millipore Corporation
Anti-NeuN, guinea pig polyclonal		1:300	ABN90P. EMD Millipore Corporation
Anti-LAMP1 antibody—lysosome marker		1:1500	ab24170, Abcam
Goat anti-rabbit IgG (H+L) highly cross-adsorbed secondary antibody, Alexa Fluor 488		1:1500	A-11034, Thermo Fisher Scientific
Goat anti-mouse IgG (H+L) cross-adsorbed secondary antibody, Alexa Fluor 555		1:1500	A-21422, Thermo Fisher Scientific
Goat anti-mouse IgG (H+L), cross-adsorbed secondary antibody, Alexa Fluor 488		1:500	A11001, Invitrogen
Donkey anti-guinea pig IgG antibody, Cy3 conjugate 555		1:750	AP193C, EMD Millipore Corporation
Isolectin GS-IB4 from Griffonia simplicifolia, Alexa Fluor® 568 conjugate		1:200	I21412, Thermo Fisher Scientific
Biotinylated peanut agglutinin lectin		1:10,000	B-1075, Vector Laboratories
Biotinylated *Maackia amurensis* lectin II		1:20,000	B-1265, Vector Laboratories
Streptavidin-horseradish peroxidase conjugate		1:1000	RPN1231-100UL, GE Healthcare Life Sciences

### Lectin blotting

Lectin blot analyses were conducted using biotinylated peanut agglutinin (PNA, Vector Laboratories, Burlington, ON, Canada), specific to carbohydrate sequence Gal-β (1–3)-GalNAc, and biotinylated *Maackia amurensis* lectin II (MAL-II, Vector Laboratories), specific for α2–3-linked sialic acid. Twenty-five micrograms of protein from each rat brain homogenate was resolved on an 8% SDS-PAGE gel and transferred to nitrocellulose membranes. Blots were blocked with 3% bovine serum albumin and then incubated overnight at 4 °C with biotinylated lectins (see Table 1 for dilutions used). Subsequently, membranes were incubated with streptavidin-horseradish peroxidase conjugate (1:1000; GE Healthcare Life Sciences, Baie-d’Urfé, QC, Canada). Lectin reactivity was detected by enhanced chemiluminescence (ECL) using the Pierce™ ECL Western Blotting Substrate (Thermo Fisher Scientific Inc., Rockford, USA). The total intensities of the stained protein bands were quantified by ImageJ software (Rasband, W.S., ImageJ, U.S. National Institutes of Health, Bethesda, Maryland, USA, https://imagej.net/, 1997–2016) and normalized for the combined intensity of protein bands on the same lane stained with Ponceau Red.

### Histochemistry and immunohistochemistry

In rats, coronal brain sections of 50 μm were cut from OCT-embedded frozen brains using a CM3050 S Microtome (Leica). For histochemical visualization of sialidase activity in the rat brain, slices were incubated with 0.2 mM sialidase substrate 1, 5-bromo-4-chloroindol-3-yl 5-acetamido-3,5-dideoxy-α-d-glycero-d-galacto-2-nonulopyranosidonic acid (X-Neu5Ac, Sigma Aldrich) at pH 4.7 in the absence or presence of pan-sialidase inhibitor 2,3-didehydro-2-deoxy-*N*-acetylneuraminic acid (DANA, 0.5 mM) for 1 h. The reaction was stopped after 60 min of incubation by addition of 1 mL of 0.4 M glycine buffer, pH 10.7. After staining, sections were rinsed in PBS and mounted on Super Frost glass slides (Thermo Fisher Scientific) using a Vectashield mounting medium (Vector Labs).

For fluorescent confocal microscopy, brain sections were blocked with goat serum containing Triton™ X-100 in PBS and incubated overnight at 4 °C with primary antibodies (Table 1). Sections were further washed with PBS, incubated with the appropriate secondary antibodies containing the nuclear staining DRAQ5 (1:400, Thermo Fisher Scientific) and after another wash with PBS, mounted on SuperFrost Plus slides, using Fluorogel (EMS, Cedarlane, Burlington, ON, Canada) or a Vectashield mounting medium (Vector Labs). In the case of the Griffonia simplicifolia I isolectin B4 (ILB4; Thermo Fisher Scientific, Table 1), which has an Alexa Fluor 568 dye conjugate, the conjugate was added during the secondary antibody incubation.

Sections were analyzed and photographed using a LSM510 Meta Laser inverted confocal microscope (Zeiss, × 63 oil objective, N.A. 1.4). Images were processed and quantified using the LSM image browser software (Zeiss) and ImageJ software.

### Glycoprotein sialylation analysis in rat brain homogenates

#### Protein extraction

Brain homogenate samples (20 mg per mL) were centrifuged at 14,000×*g* for 2 min. The 50 μL aliquots of supernatants were transferred to 1.5-mL tubes and mixed with 400 μL of 4:3:1 methanol/water/chloroform. The sample was vortexed for 20 s and centrifuged (10,000×*g*, 2 min). The upper phase was carefully removed and replaced with 200 μL of methanol. The sample was vortexed for 10 s and then centrifuged at 10,000×*g* for 2 min. The supernatant was removed, and the pellet was washed with methanol and dried under vacuum after freezing on dry ice.

#### Release and labeling of glycosylamines

The dried protein residues were mixed with 6 μL aliquots of 5% (*w*/*v*) solution of RapiGest SF in Rapid Buffer (Waters, ON, Canada) and heated at 90 °C for 3 min. The samples were cooled to ambient temperature, mixed with 1.2 μL of Rapid PNGase F and 22.5 μL of Rapid Buffer (Waters, ON, Canada), incubated at 50 °C for 5 min and cooled to ambient temperature. In each sample, glycans were labeled with RapiFluor-MS reagent and enriched using a GlycoWorks HILIC μElution Plate following the manufacturer’s protocol (Waters, ON, Canada).

#### Analysis of samples by LC-MS

For glycan profiling, liquid chromatography followed by detection by fluorescence and mass spectrometry (HPLC-FLD-MS) was performed using an Agilent 1200 SL HPLC System with a Glycan PAC AXH-1 analytical column, 2.1 × 150 mm, 1.9 μm particle size (Thermo Scientific), with precolumn thermostatted at 40 °C. A buffer gradient system composed of acetonitrile and water (96:4) as mobile phase A and 80 mM ammonium formate, pH 4.45, in water as mobile phase B was used. An aliquot of the sample was loaded onto the column at a flow rate of 0.4 mL min^− 1^ and an initial buffer composition of 90% mobile phase A and 10% mobile phase B. The column was washed for 1 min under these conditions. Glycans were eluted with a linear gradient from 10 to 20% of mobile phase B over a period of 4 min, 20 to 30% over 40 min, 30 to 45% over 5 min, and 45 to 55% over 5 min. Flow was split 1:1 between fluorescence and MS detectors. Fluorescence signals were recorded using excitation at 265 nm and emission at 425 nm. Mass spectra were acquired in a positive ionization mode using an Agilent 6220 Accurate-Mass TOF HPLC/MS system (Santa Clara, CA, USA) equipped with a dual sprayer electrospray ionization source. Mass correction was performed for every individual spectrum using peaks at m/z 121.0509 and 922.0098 from the reference solution. Mass spectrometric conditions were as follows: drying gas 10 L per min at 325 °C, nebulizer 20 psi, mass range 100–3200 Da, acquisition rate of ~ 1.03 spectra/s, fragmentor 175 V, skimmer 65 V, capillary 3800 V, instrument state 4 GHz High Resolution. Data were analyzed using the Agilent MassHunter Qualitative Analysis software package version B.07.01.

### Ganglioside composition analysis

#### Expression and purification of recombinant endoglycoceramidase (EGCase I)

Recombinant EGCase I tagged with N-terminal His tag has been expressed in *E. coli* and purified using Ni-NTA superflow column as described [34]. The activity of purified EGCase I was tested using GM3 as a substrate. One unit of EGCase I was defined as the amount of enzyme that hydrolyzed 1 μmol of GM3 per minute at 30 °C.

#### Extraction of gangliosides from rat brains

Extraction and purification of gangliosides was performed following the method of Schnaar and co-workers [35]. Essentially, rat brain homogenates (LPS or Sham treated, 10 μL aliquots each) were diluted with four volumes of ice-cold water (4 mL g^− 1^, based on tissue weight) and homogenized. Methanol was then added to a final ratio of methanol/water of 8:3. After mixing, the suspension was brought to room temperature and chloroform was added to the final ratio of chloroform/methanol/water 4:8:3 (*v*/*v*/*v*). The mixture was then subjected to centrifugation on a tabletop centrifuge (5424, Eppendorf, Germany) at 1500 rpm for 15 min. The supernatant was transferred to a fresh centrifugation tube, and 0.173 volumes of water were added. After mixing, the resulting two-phase solution was centrifuged again at 1500 rpm for 15 min. The upper phase (~ 80% of the total volume) was recovered and transferred to a fresh, capped tube. The recovered extract was then purified using a Waters Sep-Pak C18 cartridge, evaporated to dryness under a stream of nitrogen, and re-dissolved in methanol at a concentration of 20 μL/mg of original tissue weight.

#### EGCase I digestion

The final methanol extract was dried under a stream of nitrogen, and the residue was re-suspended in a 50 mM sodium acetate buffer (pH 5.2) at a concentration of 1 mg/mL of buffer. Gangliosides were then incubated for 18 h at 30 °C with 0.086 U EGCase to release the corresponding glycans.

#### Fluorescent labeling and analysis of glycans

Glycans were subsequently labeled with 20 μL of a reagent mixture (30 mg anthranilic acid, 20 mg boric acid, 45 mg sodium acetate in 1 mL of methanol) at 80 °C for 45 min. After cooling the reaction mixture, 1 mL of acetonitrile/water (97:3, *v*/*v*) was added. The mixture was vortexed and purified on a Discovery DPA-6S SPE amide column (Supelco) as described [36]. The final eluent was reduced under vacuum before further use. Labeled glycans were analyzed by LC-MS (negative-ion mode) using a normal-phase column (Accucore-150-Amide-HILIC, 2.6 μm, 2.1 × 150 mm, Thermo Fisher). The fluorescence detector was set to monitor excitation at 320 nm and emission at 420 nm, and all chromatography was performed at 40 °C. The binary solvent system followed a linear gradient with a flow rate of 0.4 mL min^− 1^ (Solvent A: 100 mM ammonium formate, pH 4.45; Solvent B: acetonitrile). Mass spectrometry (MS) was used to assign and confirm the identity of fluorescent peaks of labeled glycans. Quantification of relative glycan concentrations was done using normalized integrations from the fluorescence chromatogram.

### Statistics

Statistical analysis has been performed by Mann-Whitney or Kruskal Wallis statistical tests using GraphPad Prism. All data are presented as mean ± SD of at least two independent experiments. Statistical significance was set at *p* < 0.05.

## Results

### Postnatal inflammatory exposure causes specific induction of cerebral NEU1

The majority of lysosomal storage diseases, especially those caused by deficiencies of glycosidases, are associated with progressive neuroinflammation presumably caused by autophagy impairment and increased production of lysosomes and their exocytosis triggering activation of microglial cells (reviewed in [26, 27]). In order to test for a similar connection between neuroinflammation and lysosomal induction, we measured levels of lysosomal enzymes and protein markers in the brains of male Sprague-Dawley rat pups exposed to LPS.

**Fig. 1:**
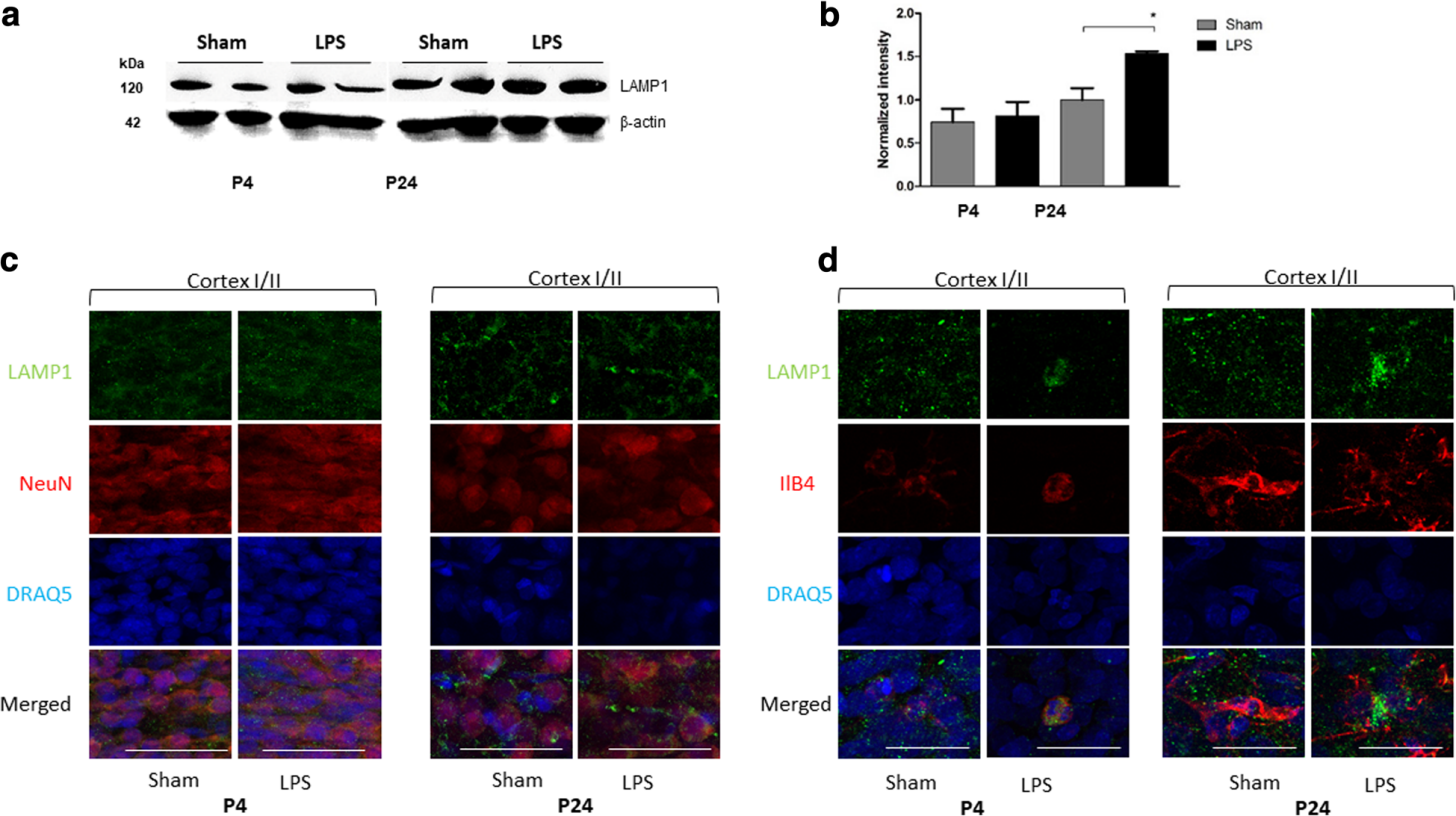
LAMP-1 protein level was increased 21 days after LPS exposure. LAMP-1 protein levels were measured by Western blot in the brain homogenates of rat pups injected with LPS (*n* = 8) and Sham (*n* = 8) 1 day (P4) and 21 days (P24) after injection, *p < 0.05 (**a**, **b**). LAMP-1 distribution in upper layers of somatosensory cortex (I–II) in neurons (NeuN-positive cells) (**c**) and microglia (ILB4-positive cells) (**d**) of LPS and Sham animals at P4 and P24 was analyzed by immunofluorescent confocal microscopy. Cortex I–II, cortical layers according to [64]

First, by Western blot, we studied the levels of the lysosome-associated membrane protein 1 (LAMP-1). Our results (Fig. 1a, b) indicated that average LAMP-1 protein levels in brain homogenates were increased following LPS exposure. Moreover, immunostaining showed that NeuN-positive neurons showed similar distribution, size and levels of LAMP-1-positive lysosomal puncta (Fig. 1c) whereas the increased levels of LAMP-1 protein were observed only in activated microglia cells positively stained for ILB4 (Fig. 1d).

**Fig. 2:**
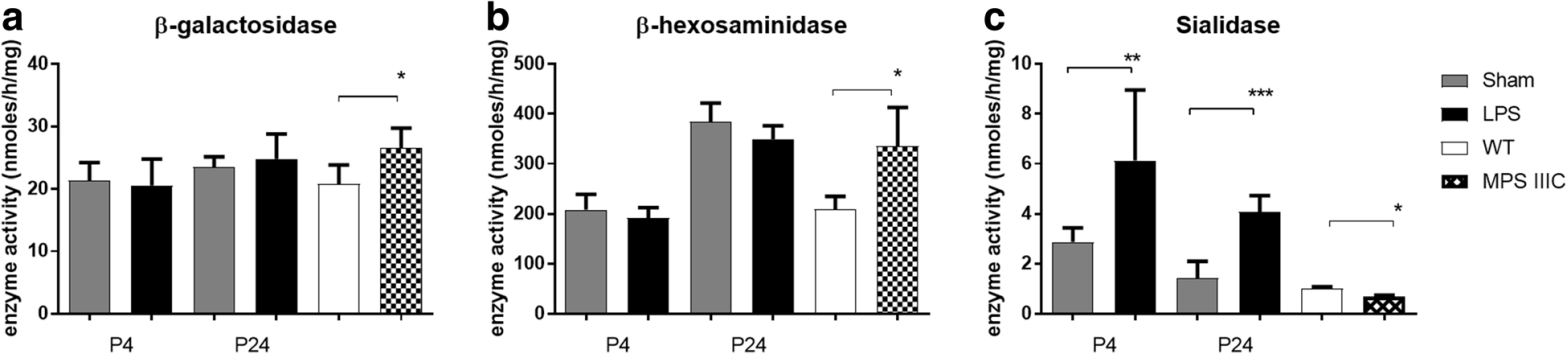
Neuraminidase activity is increased in brains of rat pups exposed to LPS. β-galactosidase (**a**), β-hexosaminidase (**b**), and neuraminidase (**c**) activities were measured in the brain homogenates of rat pups injected with LPS (*n* = 8) and Sham (*n* = 8) 1 day (P4) and 21 days (P24) after injection, and in the brain homogenates of 8-month-old MPSIIIC (*Hgsnat* KO, *n* = 4) and C57BL16 WT (*n* = 4) mice. *p < 0.05, ** *p* < 0.01, ***p < 0.001

To test if LPS treatment increased the activity of lysosomal hydrolases, we measured levels of several enzymes in the homogenates of rat brain tissues adjacent to the site of LPS or saline injections. The changes in the enzyme activity levels were compared with those induced by the lysosomal storage in the brain of the mouse model of neurological LSD, mucopolysaccharidosis IIIC (MPSIIIC) previously generated in our lab [32]. We found that, in contrast with the MPSIIIC mouse brain that showed remarkable increase of lysosomal β-galactosidase and β-hexosaminidase activities, there was no apparent differences in activities of these enzymes between LPS-exposed and Sham rat pups (Fig. 2a, b). Yet, at both P4 and P24, we saw a significantly increased acidic α-neuraminidase (sialidase) activity in the LPS-exposed rat pups (Fig. 2c). At 24 h post-injection, the neuraminidase activity (nmoles/h/mg) reached 6.13 ± 1.00 in LPS-exposed brains as compared with 2.87 ± 0.57 in control (*p* = 0.0134); at P24, the sialidase activity was increased to 4.08 ± 0.26 in LPS-exposed as compared with 1.42 ± 0.24 in control (*p* < 0.0001).

**Fig. 3:**
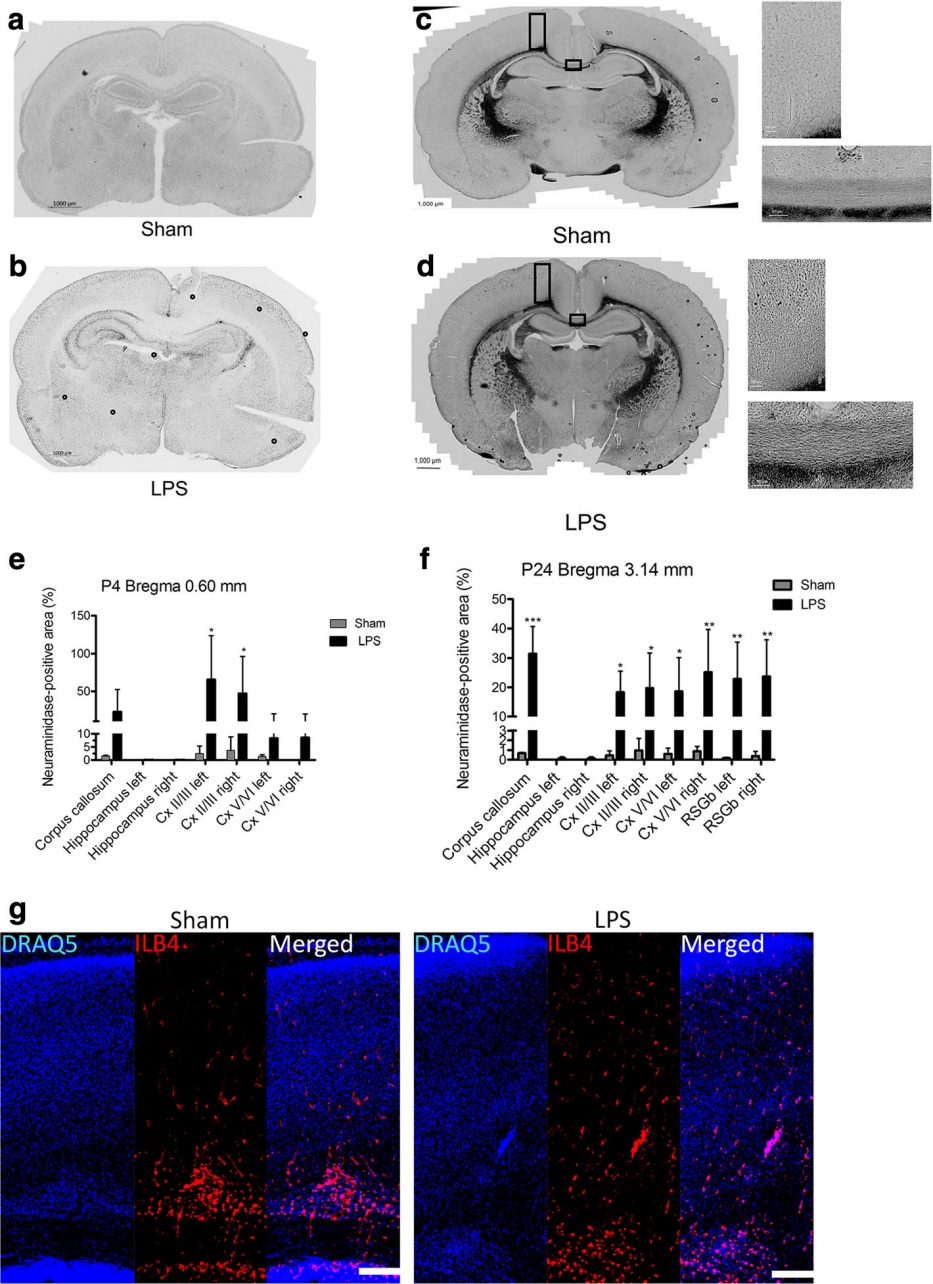
Pan-neuraminidase activity against the X-Neu5Ac substrate is increased in the cortex of LPS-injected rats and shows a pattern different from that of activated microglial cells. Panels (**a**) and (**b**) show representative coronal brain slices of Sham (*n* = 3) and LPS-injected (*n* = 3) rat pups 1 day (P4) after injection and panels (**c**) and (**d**), 21 days (P24) after injection. The bar graphs (**e**) and (**f**) show a relative size of X-Neu5Ac-stained areas (% of total areas) measured in seven different regions for P4 brains: corpus callosum, hippocampus (left and right), and the upper (layers II–III) and lower (layers V–VI) somatosensory cortices (left and right), and in nine different regions for P24 brains: corpus callosum, hippocampus (left and right), the upper (layers II–III) and lower (layers V–VI) somatosensory cortices (left and right), and RSGb (left and right). **g** Cortical distribution of activated microglia marked with ILB4 at P4 (scale bar = 200 μm) *p < 0.05, *** *p* < 0.001

To confirm the results of enzymatic assays in the tissue homogenates and to localize the areas of brain with increased neuraminidase activity, we stained coronal brain sections of the LPS-injected and Sham rats with the histochemical neuraminidase substrate X-NeuNAc. At both P4 and P24, we observed a drastic increase in staining in the LPS-exposed animals in several brain regions including periventricular areas, and superficial cortex (Fig. 3a–d). Pre-treatment of the brain sections with pan-neuraminidase inhibitor DANA almost completely blocked the staining (not shown) indicating that it was specific for sialidase activity. Quantification of the areas positive for X-NeuNAc staining at P4 in the corpus callosum, the hippocampus (left and right), and the upper (layers II–III) and lower somatosensory cortices (layers V–VI) (left and right) showed a significant increase in the corpus callosum and upper (layers II–III) somatosensory cortex (Fig. 3e). At P24, a significant increase in staining was found in the corpus callosum, in the upper (layers II–III) and lower somatosensory cortices (layers V–VI) (left and right), and in retrosplenial granular cortex (RSGb, left and right) (Fig. 3f). Such distribution of the increased neuraminidase activity differed from that of ILB4-positive activated microglia, which is highly expressed in the vicinity of the white matter (Fig. 3g).

**Fig. 4:**
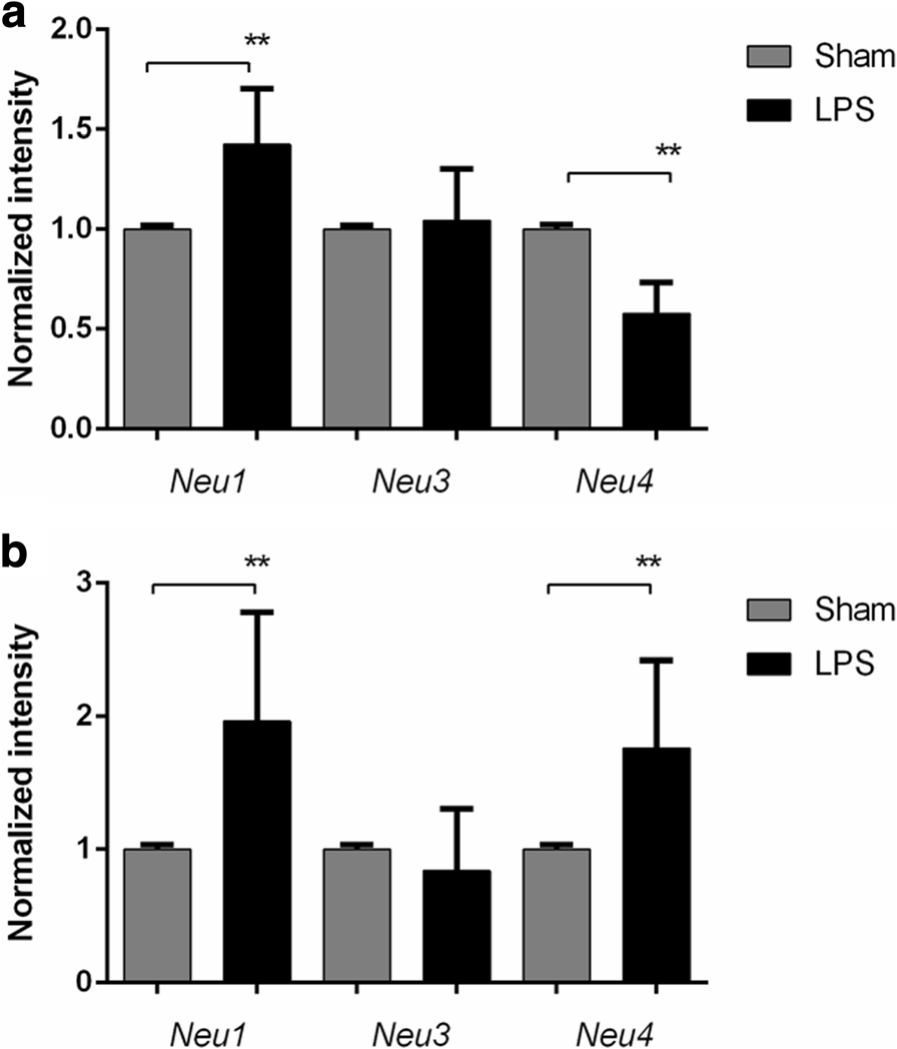
*Neu1* mRNA is increased in brains of rat pups exposed to LPS. Expression of *Neu1*, *Neu3*, and *Neu4* mRNA was measured in brain tissues of animals injected with LPS (*n* = 5) or saline (Sham) (*n* = 7) at (**a**) 24 h (P4) and (**b**) 21 days (P24) after injection. Total RNA was extracted from tissues of LPS-injected and Sham rats at P4 and P24 to analyze the expression of neuraminidases by RT-qPCR. The values were normalized for the level of control *Gapdh* mRNA. **p < 0.01

To discriminate between the three major neuraminidase isoenzymes (*Neu1*, *Neu3*, and *Neu4*) present in the rodent brain, we measured levels of the corresponding mRNAs by RT-PCR. After both 24 h and 21 days after LPS injection, a significant increase of *Neu1* mRNA in LPS-exposed animals was observed which suggested that this enzyme is responsible for the increase of the sialidase activity (Fig. 4). In contrast, the *Neu4* mRNA was significantly decreased at P4 and increased at P24, while there was no difference in the expression of the *Neu3* gene between the LPS-injected and Sham groups at both P4 and P24 (Fig. 4).

### Neu1 induction in LPS-exposed rat pups is associated with sustained desialylation of cerebral glycoproteins without detectable changes in ganglioside profiles

**Fig. 5:**
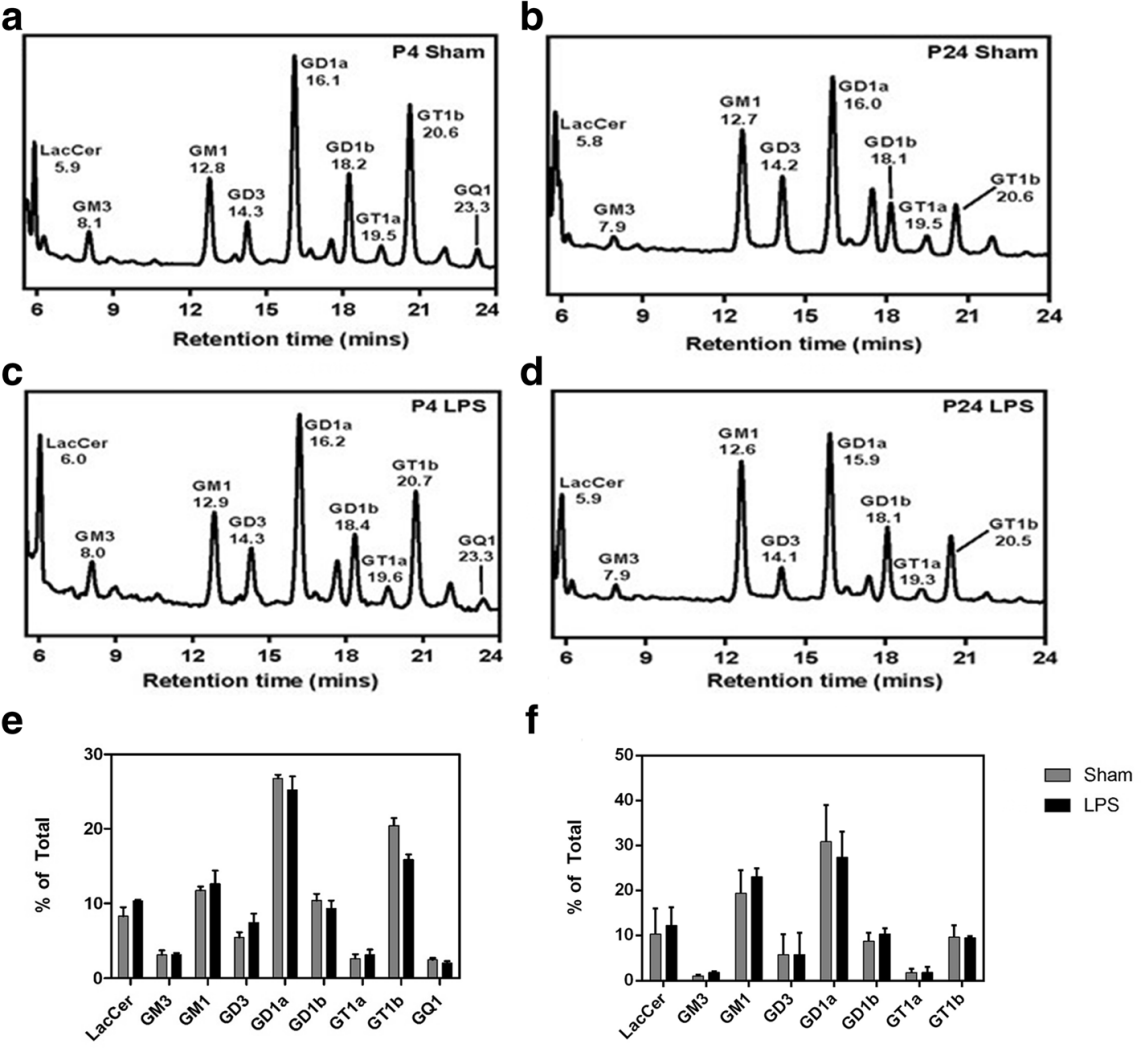
**a**-**d** Representative HPLC chromatograms of glycan chains of rat brain GSLs labelled with anthranilic acid. Panels show representative chromatograms of P4 Sham (n=2) (**a**), P24 Sham (n=2) (**b**), P4 LPS (n=2) (**c**) and P24 LPS (n=2) (**d**) samples, with fluorescence intensity (arbitrary) shown on the y-axis. **e**, **f** Levels of gangliosides measured by quantification of HPLC chromatograms. (**e**) Comparison of relative compositions of LacCer, GM3, GM1, GD3, GD1a, GD1b, GT1a, GT1b and GQ1 gangliosides in P4 LPS and Sham brains. (**f**) Relative compositions of LacCer, GM3, GM1, GD3, GD1a, GD1b, GT1a and GT1b gangliosides in P24 LPS and Sham brains. Values represent means ± S.D. of duplicate (P4) or triplicate (P24) measurements

To determine if the persistent increase in the *Neu1* expression and neuraminidase activity in the brain tissues of LPS-exposed rat pups caused any changes in sialylation of brain tissue proteins or lipids, we analyzed abundance of sialic acid residues in sialoglycoconjugates of brain tissues by LC-MS. Our results show that although the ganglioside composition of the rat brains was different between P4 and P24 samples, reflecting changes previously associated with development [37], no difference was observed between the brains of LPS-exposed and Sham animals (Fig. 5).

**Fig. 6:**
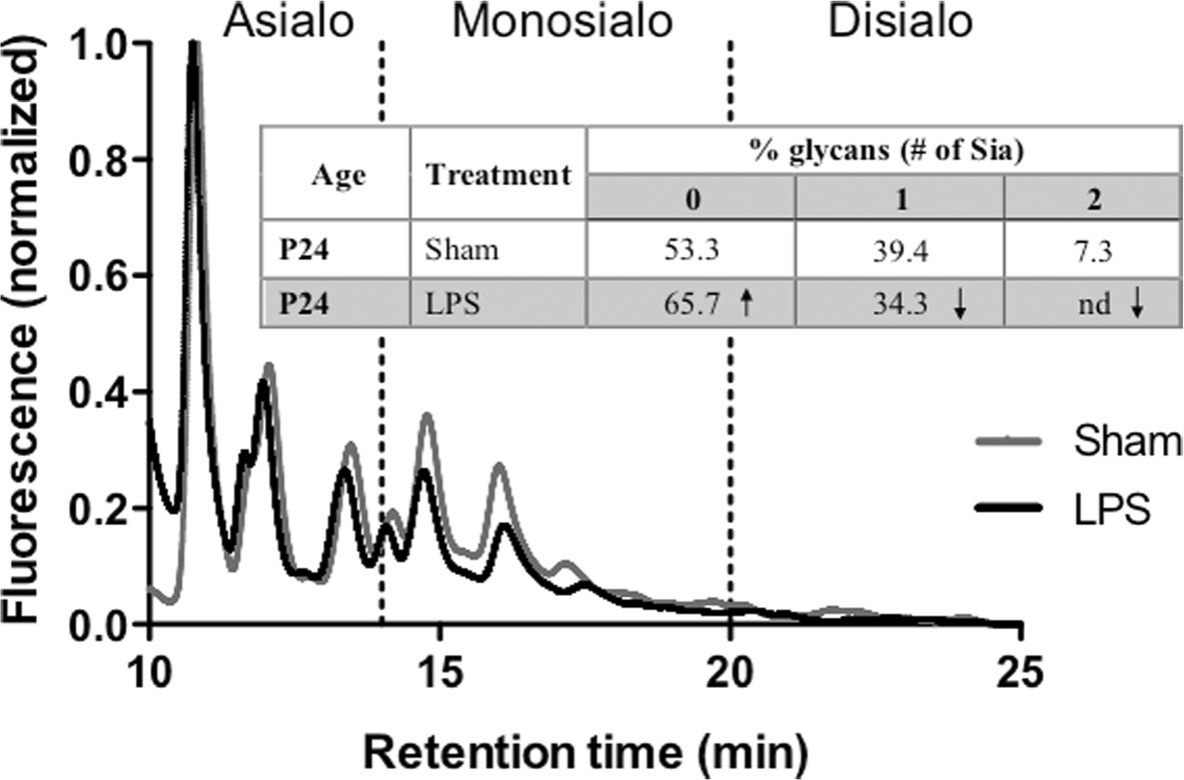
Profile of N-glycans from brain tissues of LPS-exposed and saline-treated (Sham) animals 21 days after injection. Fluorescence chromatograms of N-glycans cleaved from proteins in homogenates of brains from Sham and LPS-injected rat pups were analyzed by LC-MS after fluorescent labeling. Samples were fractionated on an anion exchange column, where glycans with higher content of sialic acid elute at later retention times. Shaded regions indicate the retention times of glycans containing 0 (< 14.5 min), 1 (14.5–20 min), and 2 (> 20 min) sialic acid residues. The traces show fluorescence intensity (arbitrary units). Individual peaks were assigned to specific glycan structures using the MS data and quantified by integrating the areas under the chromatograms (table in the inset). Figure shows representative profiles of duplicate experiments

In contrast, LC-MS analysis of N-linked protein glycans showed changes in the sialylation between the LPS-exposed and Sham animals at P24. The glycans with two or more sialic acids were completely eliminated, and the levels of monosialylated glycans reduced upon LPS treatment (Fig. 6).

**Fig. 7:**
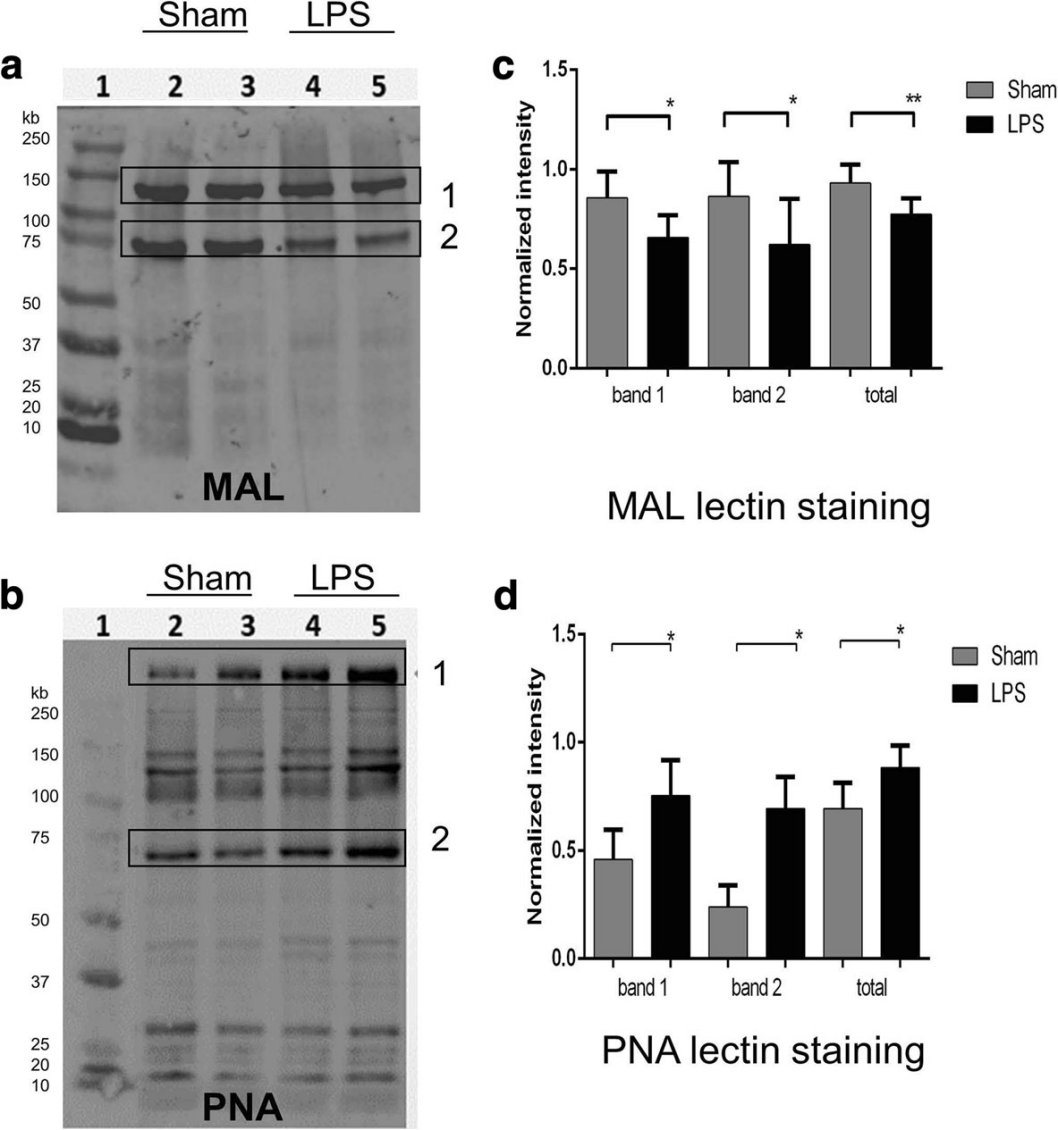
21 days after LPS injection, rat brain tissue glycoproteins show reduced staining for MAL-II lectin and increased staining for PNA lectin consistent with their desialylation. **a**, **b** Representative glycoprotein patterns of brain tissue homogenates of Sham (lanes 2–3) and LPS-injected (lanes 4–5) rat pups at P24 after injection. Following SDS-PAGE, blots were stained with biotinylated peanut agglutinin (PNA) lectin, specific for Gal-GalNAc residues and biotinylated *Maackia amurensis* II (MAL II) lectin, specific for α2–3-linked sialic acids. The bands showing apparent difference in the intensity between LPS and Sham samples are boxed. **c**, **d** Intensity of lectin-positive bands normalized to the level of total protein. For statistical analysis, five animals from each group were used; *p < 0.05, **p < 0.01

To confirm changes in the sialylation of glycoproteins in LPS-exposed brains, we analyzed total brain proteins by lectin blotting. We detected a significantly reduced MAL II and increased PNA staining of multiple protein bands consistent with the removal of the terminal sialic acid residues from the Gal-β (1–3)-GalNAc groups in the glycan chains of brain glycoproteins (Fig. 7). Together, the LC-MS analysis and lectin blotting data were consistent with an increased NEU1 activity modifying brain glycoproteins while having no effect on glycolipids.

### Specific reduction of polysialic acids on the surface of cortical neurons from LPS-treated rat pups

**Fig. 8:**
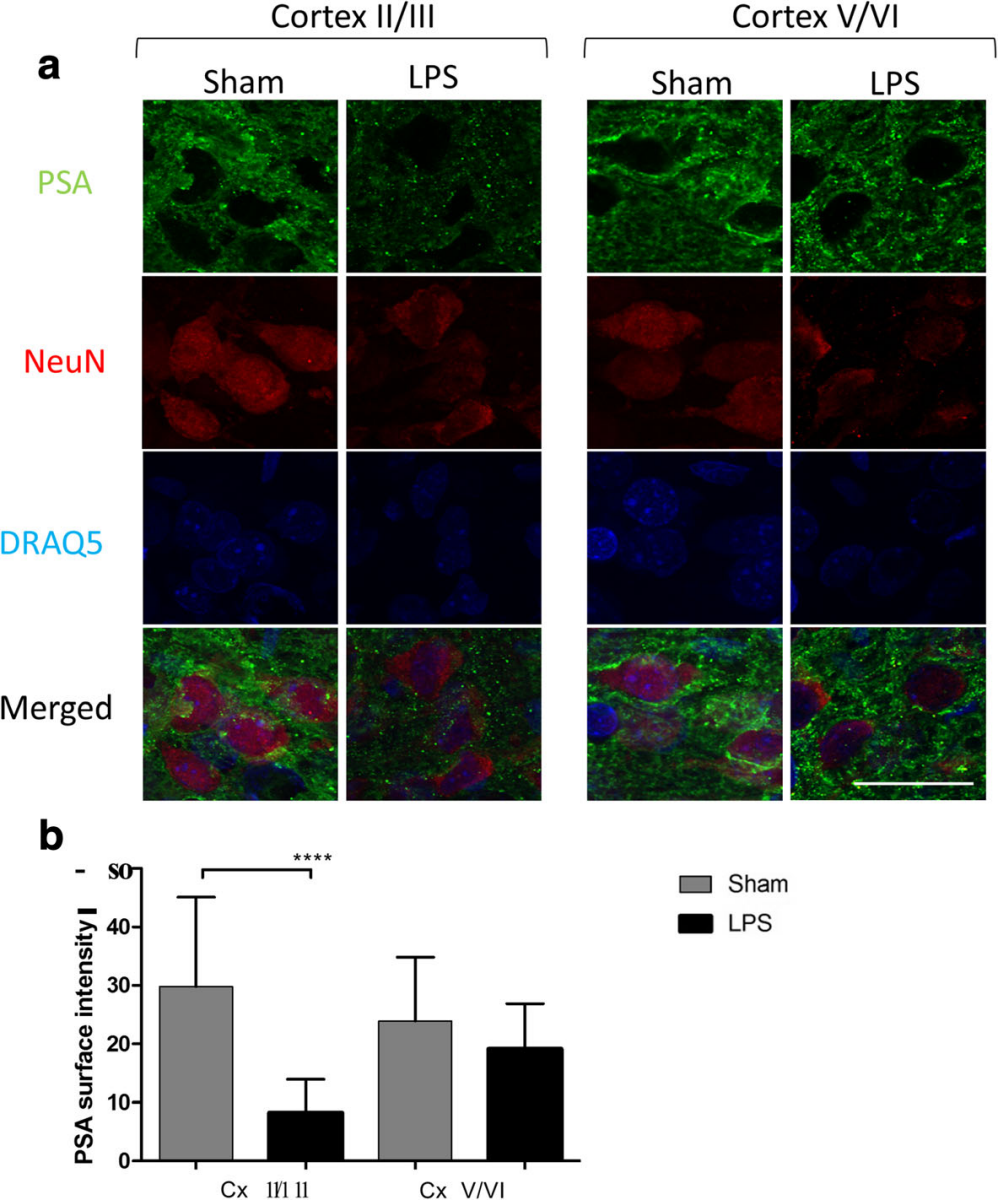
21 days after LPS exposure, upper cortical neurons show reduced PSA immunostaining. **a** Neurons with decreased PSA immunostaining are detected in the upper somatosensory cortex (layers II–III) of LPS-injected rat pups at P24 (*n* = 3) after injections as compared to Sham animals (*n* = 3). PSA, green; neuronal marker NeuN, red. Bar graphs (**b**) show average PSA immunostaining intensity in cortical neurons counted for nine adjacent 0.0042-mm^2^ sections of upper and lower layers of the somatosensory cortex. Bar represents 20 μm. ****p < 0.0001. n.s., non-significant, Cx, cortex; I–VI, cortical layers according to [64]

Modification with PSA occurring on a number of brain glycoproteins, including the neural cell adhesion molecule (NCAM), is implicated in several brain functions such as synaptic connections and plasticity as well as axonal growth and regeneration (reviewed in [11]). To detect if PSA levels are affected in the brains of LPS-exposed rat pups, we performed immunofluorescent analysis of fixed brain sections from LPS-exposed and Sham rat pups collected at P24 using monoclonal antibodies specific against PSA, counter-stained with NeuN (Fig. 8). The confocal high-resolution imaging showed that PSA was reduced in NeuN-positive neurons, while quantification of the immunostaining in multiple cortical regions revealed that, 21 days after LPS exposure, neurons in the upper cortical layers (layers II–III) have a significant reduction in PSA immunostaining (*p* < 0.0001) compared to the saline-treated rats whereas differences were not significant (*p* = 0.1081) for the lower cortical layers (V–VI) (Fig. 8).

## Discussion

Brain SGC are synthesized by a family of sialyltransferases [38] and removed by the enzymes of neuraminidase family (NEU1–4) with different tissue expression, intracellular localization, and substrate specificity. Importantly, besides catabolism of SGC, neuraminidases are involved in “trimming” sialic acid residues from glycoconjugates on the plasma membrane [39, 40]. Gangliosides and glycoproteins may also undergo rapid re-sialylation by plasma membrane-associated sialyltransferases [40]. Thus, sialylation is a dynamic modification, modulated by the interplay of sialyltransferases and neuraminidases in response to external or internal stimuli [41]. Recently, induction of neutral brain neuraminidase (presumably Neu4) was detected in rat brain hippocampus in response to memory processing [42]. Moreover, desialylation of SGC in neurons was detected during hippocampus-dependent memory formation in a contextual fear-conditioning paradigm [43]. Rare, but severe, human genetic disorders of sialic acid and SGC metabolism caused by mutations in the genes encoding enzymes and channels involved in SGC and sialic acid production, degradation, and trafficking manifest with systematic CNS involvement [44, 45]. Single nucleotide polymorphisms (SNPs) of sialyltransferase ST8SIA2/STX, involved in production of PSA, are linked with psychiatric disorders, such as schizophrenia, bipolar disorder, and autism spectrum disorders [46]. Mutations in the *NEU1* gene resulting in impaired catabolism of SGC, their lysosomal storage, and increased sialylation of cellular proteins cause defects in autophagy, accumulation of misfolded protein aggregates in the neurons, and neurodegeneration [47]. Besides, the *Neu3/Neu4* double-knockout mice and *Neu4* knockdown rats show learning impairment associated with changes in the composition of brain gangliosides [42, 48].

In the current study, we have demonstrated that an increase of brain NEU1 activity and decreased levels of sialic acids, including PSA, on neuronal glycoproteins were associated with an acute CNS inflammation injury. We show that in the rat neonatal model, LPS-induced neuroinflammation is accompanied by long-lasting (at least 21 days) increases of acidic neuraminidase activity. According to immunohistochemical staining, the highest levels of activity were observed in the periventricular areas, cortex, corpus callosum, and white matter. Previous work by Sumida et al. [49] established that microglia activated by LPS rapidly release extracellular vesicles containing NEU1, which could suggest that the increased neuraminidase activity we observe in LPS-treated rats originated from activated microglial cells. However, since in our experiments the patterns of activated microglia were different from those of neuraminidase activity, we speculate that the neuraminidase was increased in neuronal cells. The increase of pan-neuraminidase activity could be attributed to the induction of the expression of any of the three genes encoding for rat brain neuraminidases *Neu1*, *Neu3*, and *Neu4*. Although these enzymes have different substrates in vivo (NEU1 is primarily active against sialylated glycoproteins whereas NEU3 and NEU4 have preference for sialylated glycosphingolipids, gangliosides [33, 48]), all three neuraminidases are active against synthetic substrates 4MU-NANA and X-NeuNAc. Our data show that *Neu1* mRNA level (but not *Neu3*) was significantly increased at both 24 h and 21 days after LPS exposure, linking induction of *Neu1* expression with the augmented neuraminidase activity following LPS-induced inflammation. Neu4 may also play a role following the acute injury, as it was shown to be increased only at P21. Previously, we and others demonstrated that in both human and mouse cells, LPS action on TLR4 receptors caused rapid increase of surface NEU1 activity and that this activity was essential for feedback activation of TLR signaling and cytokine production [50–54]. NEU1 induction occurred rapidly (within several minutes following TLR stimulation) and was most likely caused by translocation of an endosomal/lysosomal pool of NEU1 to the plasma membranes. Our present data demonstrate that in the cerebral neurons, LPS caused a long-term induction of *Neu1* expression resulting in sustained levels of elevated NEU1 activity.

Analysis of brain sialoglycoconjugates by LC-MS and blotting with Sia-binding MAL-2 lectin and Gal-GalNAc-binding PNA lectin demonstrated significant reduction in sialylated N-linked glycans in LPS-exposed brains, suggesting that they are desialylated by overexpressed *Neu1*. In contrast, no changes were observed in composition of brain gangliosides. These results suggest that the LPS-triggered NEU1 induction does not alter sialylation of brain gangliosides consistent with our previous data showing normal ganglioside patterns in the *Neu1* knockout mice [48]. Most importantly, immunohistochemical analysis with anti-PSA antibodies demonstrated a drastic loss of PSA on cortical neurons.

Further studies are necessary to determine short- and long-term effects of these changes in the N-linked sialome on CNS function. However, it is tempting to speculate that NEU1 activity-dependent desialylation of glycoproteins on the neuronal surface may interfere with sialic acid signaling-dependent neural activities, including synaptic plasticity [55, 56], hippocampal memory [55, 56], neurotransmission, neural cell adhesion [55–57], and AMPA receptor trafficking [57, 58], and was associated with epilepsy [11, 59, 60], developmental delay, and neuropsychiatric disorders [56, 57, 61] such as schizophrenia and bipolar disorder observed in the patients with neonatal white matter injury. Interestingly, milk containing sialic acid in the form of sialyllactose given to suckling piglets was shown to increase levels of bound sialic acid in the prefrontal cortex and free sialic acid levels in the hippocampus with changes in white matter microstructure assessed by diffusion tensor imaging [62]. Moreover, feeding piglets a protein-bound source of sialic acid during early development also enhanced working memory and increased expression of genes associated with learning [63]. Thus, NEU1 modulation or sialic acid supplementation may constitute a novel therapeutic target for premature infants exposed to neuroinflammation.

## Conclusion

Neonatal cerebral LPS exposure in rat pups resulted in specific and sustained induction of Neu1 and Neu4, causing long-lasting negative changes in sialylation of glycoproteins on brain cells. In contrast, gangliosides were not altered by early inflammatory exposure. Reduction of sialylation was shown to affect neuronal layers II–III of the somatosensory cortex. Considering the important roles played by sialoglycoproteins in CNS function, we speculate that observed re-programming of the brain sialome constitutes an important part of pathophysiological consequences in perinatal infectious exposure.

## Abbreviations

EGCase I: Recombinant endoglycoceramidase
ILB4: Griffonia simplicifolia I isolectin B4
LAMP-1: Lysosome-associated membrane protein 1
LPS: Lipopolysaccharide
LSD: Lysosomal storage disorders
MAL-II: *Maackia amurensis* lectin II
MPSIIIC: Mucopolysaccharidosis IIIC
NCAM: Neural cell adhesion molecule
NEU1: Neuronal neuraminidase 1
PNA: Peanut agglutinin
PSA: Polysialic acid
SGC: Sialoglycoconjugates
TLR: Toll-like receptor
WT: Wild-type
X-Neu5Ac: 1, 5-Bromo-4-chloroindol-3-yl 5-acetamido-3,5-dideoxy-α-d-glycero-d-galacto-2-nonulopyranosidonic acid
